# Analysis of factors associated with physical activity in patients with acute pancreatitis: a cross-sectional study in China

**DOI:** 10.3389/fphys.2026.1786484

**Published:** 2026-07-29

**Authors:** Lu Zheng, Shuangyan Tu, Huiling Liu, Lihong Zhao, Xianlin Zhao, Rong Yang

**Affiliations:** 1 Department of Internal Medicine, Institute of Integrated Traditional Chinese and Western Medicine, West China Hospital, Sichuan University/West China School of Nursing, Sichuan University, Chengdu, China; 2 Department of Internal Medicine, Institute of Integrated Traditional Chinese and Western Medicine, Shang Jin Nan Fu Hospital of West China Hospital, Sichuan University, Chengdu, China; 3 Department of Neurology, West China Hospital, Sichuan University/West China School of Nursing, Sichuan University, Chengdu, China; 4 West China School of Nursing, Sichuan University, Chengdu, Sichuan, China; 5 West China Center of Excellence for Pancreatitis, Institute of Integrated Traditional Chinese and Western Medicine, West China Hospital, Sichuan University, Chengdu, China

**Keywords:** acute pancreatitis, cross-sectional study, education programs, international physical activity questionnaire, physical activity

## Abstract

**Objectives:**

To assess physical activity (PA) levels and associated factors in patients with acute pancreatitis (AP) in Western China.

**Methods:**

This cross-sectional study was conducted from July 2021 to June 2023 at a tertiary hospital in Sichuan Province. A total of 408 adult patients with AP were enrolled. PA levels and sedentary time (ST) were assessed using the long-form International Physical Activity Questionnaire. Sociodemographic, lifestyle, and clinical data were collected. Multivariate logistic regression was used to identify factors associated with PA levels.

**Results:**

Low physical activity (LPA) was observed in 74.3% of participants. Daily ST ranged from 60 to 600 minutes, with a mean of 317.57 ± 122.42 minutes. Screen time accounted for 68.3% of sedentary behaviors. Higher PA levels were associated with shorter ST. Non-alcoholic fatty liver disease, biliary tract disease and three or more previous AP episodes were associated with LPA. Secondary education was associated with higher PA.

**Conclusion:**

Physical inactivity is common among AP patients. Sedentary time, NAFLD, biliary tract disease, education level, and AP recurrence are key determinants.

## 1 Introduction

The global prevalence of acute pancreatitis (AP is a significant public health issue and has increased markedly in recent decades due to social development and rising living standards. The global pooled incidence of AP is approximately 34 cases per 100,000 people per year (Petrov and Yadav, 2019), whereas in China the current annual incidence of AP is lower at 23.4 cases per 100,000 people (Pang et al., 2018). The number of patients with AP generally increases with age, particularly in older populations and is showing an upward trend (Jiang et al., 2023). In addition, the incidence of hypertriglyceridemic AP has increased significantly in younger individuals (Fan et al., 2025). This condition is notable for its severity and recurrence (Gagyi et al., 2024) and has surpassed alcohol as the second leading cause of AP in China (Carr et al., 2016). Due to the evolving nature of pancreatitis, many sequelae of AP persist long after the initial clinical symptoms have subsided. These sequelae include exocrine pancreatic insufficiency, bone metabolic changes, and endocrine abnormalities, particularly diabetes, which can damage organs such as the retina, cardiovascular system, and kidneys. These complications are also associated with long-term psychological stress, economic burden, and loss of productivity (Xu et al., 2025; Lin et al., 2026; Sikora Kessler et al., 2026). According to the Global Burden of Disease Guidelines (Ouyang et al., 2020), the key risk factors for AP include demographic and socioeconomic factors, gallstones, alcohol and tobacco use, obesity, and metabolic problems. These sequelae not only impair the daily functioning of patients but also increase healthcare utilization and societal costs. This highlights the need for comprehensive management strategies that go beyond acute symptom control. In recent years, dietary habits and physical activity (PA) have received increasing attention as potential factors that contribute to AP. Poor dietary quality, characterized by diets high in refined sugars, sweets, fats, or processed meats, and low vitamin C intake, contribute synergistically to the development of AP. Diet-related obesity and hyperlipidemia are also significant risk factors for AP (Mao et al., 2023; Souto et al., 2025; Wang et al., 2025).

Existing evidence confirms that PA is beneficial for health, with appropriate exercise contributing to the prevention and treatment of cardiovascular disease, psychiatric disorders, metabolic diseases, pulmonary diseases, musculoskeletal disorders, and cancer (Dibben et al., 2024). Several recent studies in the Chinese population have also reported that total PA is associated inversely with AP and various hepatobiliary diseases (Pang et al., 2018; Pang et al., 2021). However, there is evidence that 27.5% of adults are physically inactive worldwide, with this proportion increasing over time, particularly in high-income countries (Guthold et al., 2018). This trend is concerning due to physical inactivity being a modifiable risk factor that contributes to the development of numerous chronic conditions, including those that exacerbate the burden of AP. In the context of AP, the lack of PA may worsen metabolic disturbances and promote disease recurrence. This rising trend is also observed in more economically developed regions in China, where socio-demographic factors influence regular exercise rates (Su et al., 2023). Research in Finland has indicated that low PA and high sedentary behavior lead to substantial and significant losses in societal costs (Kolu et al., 2022). Of the sedentary behavioral patterns of patients with pancreatitis, increased leisure screen time raises the risk of AP significantly, thereby exacerbating the associated health burden (Ling et al., 2023). These findings indicate that there is an urgent need to address the lack of PA by prioritizing the implementation of targeted PA promotion strategies that are based on an understanding of the specific groups characteristics.

Previous studies have investigated the prevalence of PA in the general population in China, with the results indicating that factors such as gender, education, marital status, income, and dietary habits may correlate with the level of PA (Tu et al., 2019; Wang et al., 2022). However, there is limited evidence specific to patients with AP in Western China, where development of the disorder is uneven. Sichuan province, a representative province in Western China, is known for its unique culture and cuisine. In addition, urbanization in the region has resulted in the number of patients with AP related closely to lifestyle has increased markedly. Therefore, there is an urgent need in Sichuan Province to update the measurement of the prevalence of PA and its associated risk factors in the growing population of patients with AP. This would provide crucial evidence to support the promotion of PA programs within this population. Moreover, given the rapid urbanization and lifestyle changes in Western China, the findings from Sichuan Province may serve as a reference for other regions undergoing similar transitions. This would help to inform public health policies that include lifestyle modifications with the aim of reducing the burden of AP.

The current study offers novel insights into the frequency and key determinants of PA in patients with AP by identifying essential variables that contribute to PA levels. By bridging existing gaps in the data, our findings will contribute to the growing evidence supporting the need for individualized exercise prescriptions in this population. Individualized exercise prescriptions, tailored to the patients’ clinical status, physical capabilities, and personal preferences, may enhance adherence and maximize health benefits, ultimately improving long-term outcomes for AP patients.

## 2 Methods

### 2.1 Study design and population

This cross-sectional study was conducted at the West China Hospital of Sichuan University in the Sichuan Province. The participants were selected using “convenience sampling” of adults with AP with a confirmed age between July 2021 and June 2023, who met the diagnosis for AP based on the 2012 revised Atlanta classification criteria (Banks et al., 2013). Patients with either no formal education, communication difficulties, an inability to complete the questionnaires, a history of psychiatric illness or use of psychotropic drugs, comorbid malignancies, or pregnancy were excluded from the study. The study was approved by the Ethics Committee of West China Hospital of Sichuan University (approval number: 20211691). Written informed consent was obtained from all the respondents who were advised of the purpose of the study, procedures, and an assurance of the confidentiality of personal information.

### 2.2 Sample size

The sample size included 294 respondents. The sample size calculation was based on an estimated prevalence of moderate-to-high PA (MPA/HPA) of 25.7%, derived from a pre-survey of a pilot sample at our center. Based on 5% precision and a confidence interval of 95%, we calculated the required sample size using the single proportion sample size formulae, [ $n=\frac{Z_{\alpha }^{2}\times P\times (1-P)}{d^{2}}$]. The survey questionnaire was distributed to 500 participants, with a response rate of 81.6% (408 respondents). This number of participants was considered sufficient for the analysis. Of the participants who responded, 71.8% were males and 18.2% females, with a mean age of 41.6 years.

### 2.3 Data collection

The data for general demographic characteristics and lifestyles were included in a standard questionnaire, and included gender, age, education, smoking habits, alcohol consumption, dietary behavior in the past year, medical history, and activities of daily living and leisure time. Educational level was classified into none, elementary (≤ 6 Y), secondary (7–12 Y), or university (> 12 Y). Marital status was classified into unmarried (including never married, divorced, or widowed) or married. This classification acknowledged that divorced and widowed individuals may have distinct psychosocial characteristics, although their small number precluded separate analysis. The height (m) and weight (kg) of the participants were measured in order to calculate the body mass index (BMI, kg/m^2^). According to the Guidelines for Prevention and Control of Overweight and Obesity in Chinese adults (Chen and Lu, 2004), underweight is defined as BMI < 18.5 kg/m^2^, normal weight as 18.5 kg/m^2^ ≤ BMI < 24 kg/m^2^, overweight as BMI ≥ 24 kg/m^2^, and obesity as BMI ≥ 28 kg/m^2^.These classifications were slightly different from those used in the World Health Organization (WHO) BMI criteria (WHO Consultation on Obesity (1999: Geneva, Switzerland) and World Health Organization, 2000). We also reviewed the patients’ electronic medical records to obtain clinical data, which included combined diseases, laboratory data, and imaging examinations. A gastroenterologist diagnosed NAFLD based on a fiberoscan examination. Interpretation of the results of the liver fibrosis assessment were based on the METAVIR score, with the results expressed in kilopascals (Kpa). The Controlled Attenuation Parameter (CAP) test was used to assess steatosis, with the results reported in dB/m (Foucher et al., 2006; Ebrahimi-Mousavi et al., 2022). We minimized bias by ensuring anonymity and confidentiality throughout the study. The content and structure of the survey instrument tool used in the study were validated. In addition, we validated the indicators of the clinical characteristics using the hospital information system.

### 2.4 Assessment of physical activity and sedentary time

Physical activity levels and sedentary time were assessed by requesting the participants to report their daily PA using the International Physical Activity Questionnaire (IPAQ) (Craig et al., 2003). Since its development, the IPAQ has been translated into many different languages and researched extensively that has shown the versions in different countries are valid and have good reliability (Kim et al., 2013), with the long form Chinese version of IPAP having a reliability coefficient ranging from 0.67 to 0.93 (Macfarlane et al., 2011; Ren et al., 2017). To ensure accurate recall of specific exercise activities and periods, we conducted a pre-survey that showed significantly lower PA levels in AP patients, thereby making the short-form IPAQ unsuitable. Therefore, we used the long-form IPAQ consisting of 27 questions to assess PA levels and sedentary time. Sedentary behavior was defined as total time spent sitting, including specific behaviors such as sitting or lying for reading, studying and watching television, or sitting at school or during transportation in hours per week (Tremblay et al., 2017). All the time spent each week in occupational and nonoccupational activities was collected to calculate the metabolic equivalent of task hours per week (MET-h/w), which represented the product of the number of hours spent each week participating in each activity. According to the IPAQ scoring scheme, the PAs were categorized into the following three levels based on the frequency and intensity of weekly PA and the metabolic equivalent of tasks (METs) of the participants: low physical activity (LPA), moderate physical activity (MPA), and high physical activity (HPA) (Subramaniam et al., 2019). The total MET-min/week was calculated as: walk (METs*min*days) + moderate (METs*min*days) + vigorous (METs*min*days). We provided the participants with clear instructions for completing the questionnaire and also trained the enumerators using a standardized process for conducting the face-to-face, one-to-one surveys.

### 2.5 Assessment of smoking, drinking, and dietary behaviors

Daily tobacco use was measured by the Fagerstrom test for nicotine dependence (FTND), which has been validated widely by researchers in China and abroad (Guo et al., 2023). Alcohol intake was calculated using the Alcohol Use Disorders Identification Test (AUDIT) questionnaire for beer, wine, and liquor, and was converted to total grams (g) (12.8 g of ethanol for 360 mL (12 oz) of regular beer, 11.3 g for 360 mL (12 oz) of light beer, 11.0 g for 120 mL (4 oz) of wine, and 14.0 g for 45 mL (1.5 oz) of liquor) (Peila et al., 2022). Alcohol weekly intake (g/wk) was categorized as either abstinence, < 140, 140 < 420, or ≥ 140 (g/wk). The dietary behavior survey used a semi-quantitative food frequency questionnaire (FFQ) validated in the Shanghai cohort study in order to obtain information on common individual food consumption, with habitual dietary intake assessed using face-to-face interviews carried over the past year. Comparison of FFQ with multiple 24-hour dietary recalls showed that the dietary frequency questionnaire had high validity and repeatability, with the correlation coefficients of the main food groups ranging between 0.41-0.72 (Shu et al., 2004; Villegas et al., 2007). Each food item or group of food items was followed by a question on the amount consumed per unit of time (daily, weekly, monthly, yearly, or never).

### 2.6 Statistical analysis

All the data collected were recorded twice in Epidata 3.1 to ensure authenticity and accuracy. The statistical analyses were performed using SPSS 26.0 (SPSS Inc., Chicago, IL, USA). Descriptive statistics, including counts, percentages, median (quartile), and 95% confidence intervals (CI), were used to describe the data. The Mann-Whitney or Kruskal-Wallis test was used for comparison of quantitative variables between groups, while the chi-square test was used for comparison of the categorical variables. Non-parametric tests were selected due to the quantitative variables not satisfying the normality and homogeneity of variance assumptions, as confirmed by the Shapiro-Wilk test and Levene’s test, respectively. Statistically significant sociodemographic and health-related variables in the univariate analysis were added as independent variables in a multiple logistic regression analysis that was used to identify factors that influenced the population’s PA level, and also to verify whether the inclusion of independent variables was statistically significant. Statistical significance was determined using a two-sided test, with a *p*-value of < 0.05 considered significant. We collected data on potential confounding variables such as age, gender, education, marital status, health insurance, and income, and then adjusted these variables in the multiple linear regression analyses. In addition, we carefully reviewed the collected data and excluded the questionnaires with missing values, while those with doubtful results were investigated clinically again to ensure the authenticity of the results.

Multi-class logistic regression analysis was used to identify the factors that affected the level of PA in patients with AP, and to verify whether the inclusion of independent variables was statistically significant. Since screening variables in multivariate models based only on univariate *P*-values may lead to type II errors, we established two models constructed as follows. Model 1 was adjusted for variables that showed a significant association (*p* < 0.05) with PA in the univariate analysis, which incorporated one complication. To further investigate the comprehensive impact of comorbidities, Model 2 was expanded to include four additional complications. These indicators were retained in the final model irrespective of their univariate *p*-values in order to control for a potential clinical confounding effect and to evaluate their independent contribution to PA patterns. A p value < 0.05 was considered statistically significant.

## 3 Results

### 3.1 Sociodemographic characteristics

**Figure 1:**
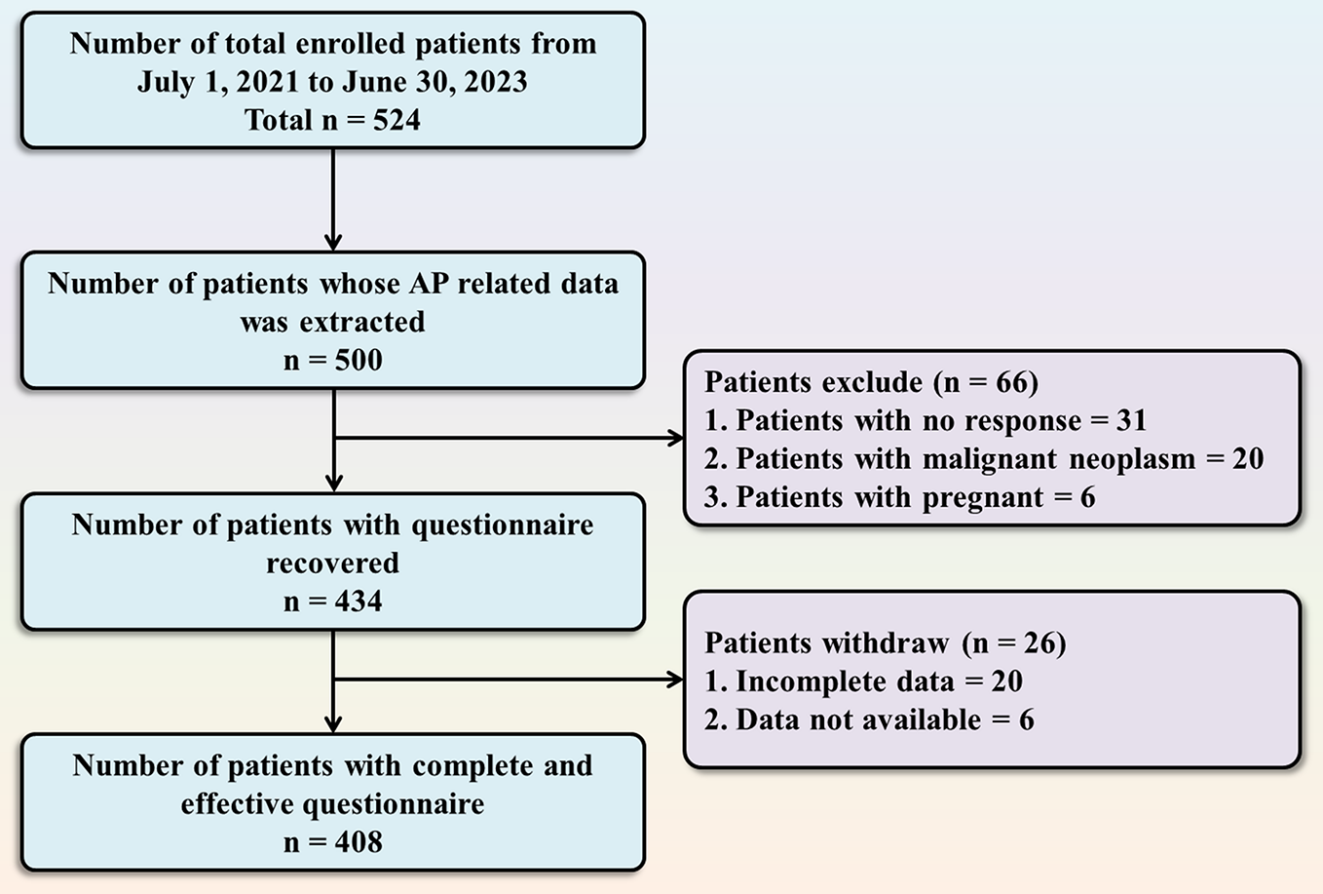
Flow diagram illustrating the participant inclusion process.

Of the 524 eligible patients identified initially, 116 were excluded for the following reasons: incomplete questionnaire responses (n = 31), diagnosis of malignant neoplasm or pregnancy (n = 26), early study termination due to transfer or discharge (n = 20), missing clinical data required for AP diagnosis confirmation (n = 24), and incomplete PA assessment (n = 6). Therefore, 408 participants with completed questionnaires, anthropometric measurements, radiological examinations, and blood tests were included in the final analysis, resulting in a response rate of 81.6% (**Figure 1**). **Table 1** shows that the participants in the study were aged between 18–69 years with a mean age (SD) of 41.6 ± 11.3. Of those participants, 239 (58.6%) were between the ages of 18–44 years and 169 (41.4%) aged 45 years or older. More than one-half of the participants were males (71.8%), lived in urban areas (69.9%), had secondary or above education (89.0%), or had LPA (74.3%). In addition, BMI calculated from on-site height and weight measurements showed that 58.3% of the participants were overweight or obese. We observed that the majority of participants had LPA (74.3%), with only 25.7% achieving MPA or HPA. The three PA levels were significantly different in the education group (χ^2^ = 10.247, *p* < 0.05), with the distribution of LPA being 57.8%, 74.7%, and 78.4% for the ‘none and elementary’, ‘secondary’, and ‘university’ education levels, respectively. In the occupation group (χ^2^ = 22.189, *p* < 0.05), the proportion of LPA was highest among ‘staff of agencies and institutions’ (85.7%) and lowest among ‘agricultural laborers’ (48.6%); and the living area group (χ^2^ = 7.285, p < 0.05), with the prevalence of LPA being 65.9% in rural areas compared to 77.9% in urban areas.

**Table 1:** Basic conditions of social demography and physique characteristics grouped according to PA level.

Variables	PA level			χ^2^ /F	*P*
	High (n = 40)	Moderate (n = 65)	Low (n = 303)		
Total					
Male, n (%)	31 (10.5%)	40 (13.7%)	222 (75.8%)	4.346	0.114
Female, n (%)	9 (7.8%)	25 (21.7%)	81 (70.5%)		
Age, years					
18-44	23 (9.6%)	33 (13.8%)	183 (76.6%)	2.065	0.356
≥ 45	17 (10.1%)	32 (18.9%)	120 (71.0%)		
Ethnic groups, n (%)					
Han	39 (10.2%)	60 (15.6%)	285 (74.2%)	1.213	0.545
Minority	1 (4.2%)	5 (20.8%)	18 (75%)		
Insurance, n (%)					
Uninsured	9 (12.2%)	11 (14.8%)	54 (73.0%)	0.598	0.742
Insured	31 (9.3%)	54 (16.2%)	249 (74.5%)		
Marital status, n (%)					
Unmarried	8 (14.3%)	10 (17.9%)	38 (67.9%)	1.84	0.399
Married	32 (9.1%)	55 (15.6%)	265 (75.3%)		
Education, n (%)					
None and Elementary (≤ 6 Y)	5 (11.1%)	14 (31.1%)	26 (57.8%)	10.247	0.036
Secondary (7–12 Y)	22 (10.9%)	29 (14.4%)	150 (74.7%)		
University (> 12 Y)	13 (8.0%)	22 (13.6%)	127 (78.4%)		
Occupation, n (%)					
Staff of agencies and institutions	2 (9.5%)	1 (4.8%)	18 (85.7%)	22.189	0.014
Corporate employees	5 (5.6%)	14 (15.6%)	71 (78.7%)		
Commercial and service industry Personnel	14 (10.1%)	25 (18.1%)	99 (71.8%)		
Agricultural laborer	8 (22.9%)	10 (28.6%)	17 (48.6%)		
Unemployed/Pensioner	3 (5.2%)	10 (17.2%)	45 (77.6%)		
Other job	8 (12.1%)	5 (7.6%)	53 (80.3%)		
Monthly income per capita (RMB, yuan)					
≤2999	12 (15.8%)	15 (19.7%)	49 (64.5%)	5.549	0.235
3000-4999	15 (8.8%)	25 (14.7%)	130 (76.5%)		
≥ 5000	13 (8.0%)	25 (15.5%)	124 (76.5%)		
Living area					
Rural	18 (14.6%)	24 (19.5%)	81 (65.9%)	7.285	0.026
Urban	22 (7.7%)	41 (14.4%)	222 (77.9%)		
Obesity					
Normal and underweight (BMI < 24.0, kg/m^2^)	20 (11.8%)	22 (12.9%)	128 (75.3%)	2.82	0.244
Overweight/Obesity (BMI ≥ 24.0, kg/m^2^)	20 (8.4%)	43 (18.1%)	175 (73.5%)		
Sedentary time	232.5 (82.858) 3**	304.62 (108.844) 1**	331.58 (124.93) 1**	3.974	< 0.001

PA, Physical Activity; BMI, body mass index.

### 3.2 Sedentary time and behavior

**Figure 2:**
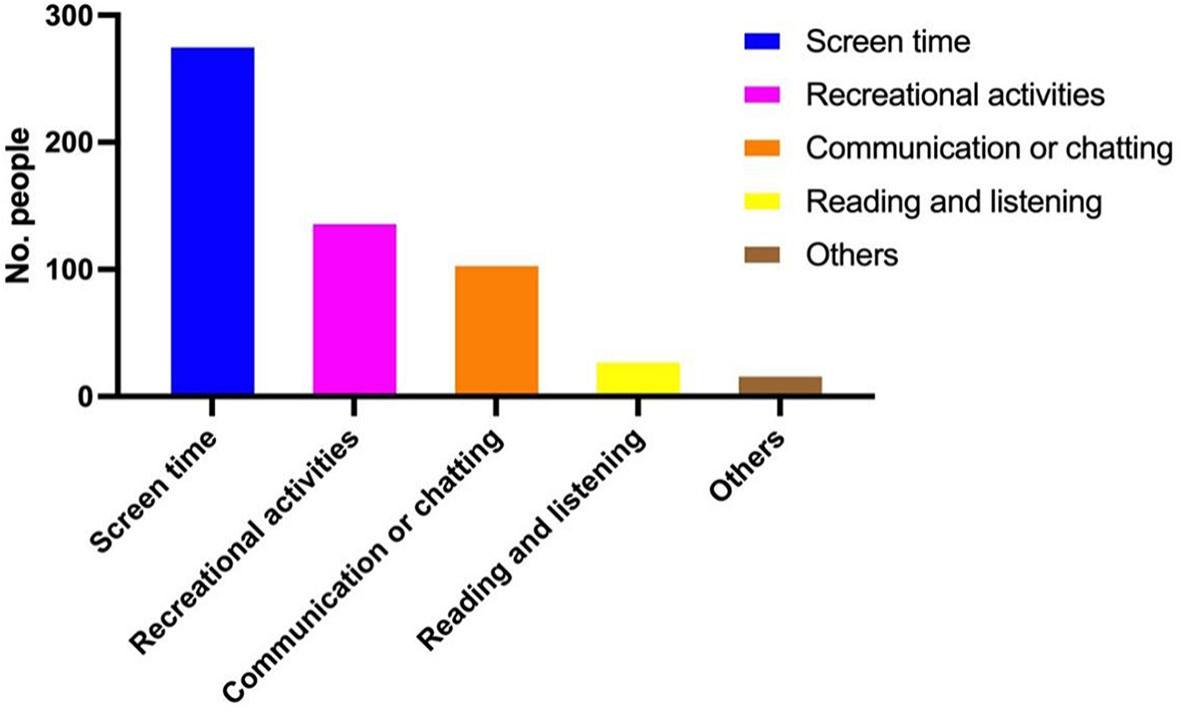
Column chart of the components of daily sedentary behaviors according to ordinary behavior.

The daily sedentary time (ST) of the participants ranged from 60 to 600 min (mean 317.57 ± 122.417 min). The mean daily ST of participants with HPA levels was significantly lower (232.5 ± 82.858 min) compared to that of participants in the other PA level groups (F = 3.974, *p* < 0.001, MPA 304.62 ± 108.844 min, LPA 331.58 ± 124.93 min). **Figure 2** shows that among the various sedentary behaviors, screen time had the highest and most significant percentage (68.3%), followed by recreational activities such as playing mahjong and card games (34.3%), and communication or chatting (26.2%). Only 7.6% of participants self-reported ST spent on reading and listening and other activities (5%). We also conducted a subgroup analysis based on the median screen time (4 h/d) of the study population. The results showed that there was a significant correlation between PA level and screen time (**Supplementary Table 1**).

### 3.3 Smoking, drinking, and dietary behaviors

The study described the level of PA according to the quantity of tobacco and alcohol used (**Supplementary Figures A, B**). Overall, more than half of the participants were current smokers or consumed alcohol. The group of participants with LPA contained the largest number of non-smokers (72.1%), while the number of smokers and non-smokers was comparable in participants with LPA or HPA. In addition, we observed a tendency for higher use of tobacco to be associated with lower levels of PA (*p* > 0.05). Similarly, participants with LPA had the largest percentage of abstainers (73.9%).

The results in **Table 2** showed that participants with LPA demonstrated a preference for meat and fruit, but also had the highest number of non-milk drinkers. Generally, the lower the level of PA, the higher the number of participants who consumed meat and fruit, and conversely a higher number of participants who refused dairy intake.

**Table 2:** Health conditions and health-related behaviors in AP patients grouped according to physical activity level.

Variables	PA level			χ^2^	*P*
	High (n = 40)	Moderate (n = 65)	Low (n = 303)		
Hypertension	4 (8.5%)	7 (14.9%)	36 (76.6%)	0.165	0.921
Diabetes	9 (10.5%)	14 (16.3%)	63 (73.2%)	0.072	0.965
Biliary tract disease	9 (9.9%)	20 (22.0%)	62 (68.1%)	3.282	0.194
NAFLD	5 (3.9%)	15 (11.5%)	110 (84.6%)	11.971	0.003
No. previous AP attacks					
0	22 (10.7%)	37 (18.0%)	146 (71.2%)	16.938	0.01
1	7 (9.7%)	13 (18.1%)	52 (72.2%)		
2	8 (21.1%)	7 (18.4%)	23 (60.5%)		
≥ 3	3 (3.2%)	8 (8.6%)	82 (88.2%)		
Cigarette equivalents/day					
Never smokers	20 (9.1%)	41 (18.8%)	158 (72.1%)	4.375	0.626
≤ 20 cigarettes/day	14 (10.9%)	14 (10.9%)	100 (78.2%)		
21–30cigarettes/day	4 (10.0%)	7 (17.5%)	29 (72.5%)		
≥ 31 cigarettes/day	2 (10.0%)	2 (10.0%)	3 (80.0%)		
Alcohol weekly intake (g)					
Abstainers	25 (10.7%)	36 (15.4%)	173 (73.9%)	4.493	0.61
< 140 g	2 (3.7%)	12 (22.2%)	40 (74.1%)		
140g to < 420 g	7 (12.1%)	7 (12.1%)	44 (75.8%)		
≥ 420 g	6 (9.7%)	10 (16.1%)	46 (74.2%)		
Dietary preferences meat ≥ 150 g/day)	20 (10.3%)	34 (17.4%)	141 (72.3%)	0.801	0.67
Consumes ≥ 1 fruit daily	35 (9.3%)	60 (15.9%)	283 (74.9%)	1.818	0.403
No dairy intake	5 (5.2%)	12 (12.5%)	79 (82.3%)	4.721	0.094

NAFLD, Non-alcoholic fatty liver disease.

Acute pancreatitis, AP; Physical activity, PA; Nonalcoholic fatty liver disease, NAFLD.

### 3.4 Clinical characteristics

**Table 2** shows that 84.6% of patients with NAFLD had LPA, while only 3.9% had HPA, with this difference being statistically significant (*p* < 0.01). Among patients with three or more previous episodes of AP, LPA was most frequent (88.2%), with this difference also being statistically significant (*p* < 0.05). No significant between-group differences in PA were observed following stratification by AP severity and etiology (**Supplementary Table 2**).

### 3.5 Analysis of influencing determinants

Multi-class logistic regression analyses were performed to identify the factors associated independently with PA levels (**Tables 3**, **4**).

**Table 3:** Multivariate logistic regression analysis of factors affecting the level of PA of patients with AP (model 1).

Variables	Low-level PA vs. high-level PA			Moderate-level PA vs. high-level PA			Low-level PA vs. moderate-level PA		
	B	OR	(95%CI)	B	OR	(95%CI)	B	OR	(95%CI)
(Intercept)	1.354			2.562			-0.968		
NAFLD	-1.402	0.246**	0.086-0.702	-0.873	0.418	0.108-1.609	-0.47	0.625	0.324-1.207
No. previous AP attacks 0	Reference			Reference			Reference		
1	0.241	1.273	0.466-3.479	-0.291	0.747	0.207-2.695	-0.049	0.952	0.453-2.001
2	1.102	3.009	0.997-9.082	0.702	2.018	0.489-8.327	0.061	1.063	0.397-2.846
≥ 3	-1.537	0.215*	0.057-0.817	-1.075	0.341	0.065-1.793	-0.971	0.379*	0.162-0.885
None and Elementary (≤ 6 Y)	Reference			Reference			Reference		
Secondary (7–12 Y)	0.027	1.027	0.277-3.816	1.932	6.903*	1.481-32.187	-0.857	0.424*	0.183-0.984
University (> 12 Y)	0.269	1.308	0.308-5.56	1.437	4.206	0.777-22.769	-0.861	0.423	0.165-1.085
Staff of agencies and institutions	Reference			Reference			Reference		
Corporate employees	-0.551	0.576	0.089-3.741	-1.856	0.156	0.009-2.691	1.142	3.132	0.37-26.539
Commercial and service industry personnel	0.308	1.361	0.246-7.535	-1.176	0.309	0.021-4.597	1.474	4.368	0.537-35.536
Agricultural laborer	0.755	2.127	0.260-17.435	-1.096	0.334	0.016-7.063	1.74	5.699	0.559-58.089
Unemployed/Pensioner	-0.942	0.39	0.049-3.094	-1.674	0.187	0.009-3.865	1.182	3.26	0.367-28.977
Other job	0.407	1.502	0.222-10.154	0.837	2.31	0.112-47.658	0.486	1.625	0.166-15.953
Rural	Reference			Reference			Reference		
Urban	-0.805	0.447	0.179-1.115	-0.421	0.656	0.219-1.963	-0.127	0.88	0.435-1.781
Sedentary time	-0.01	0.99**	0.986-0.994	-0.011	0.989**	0.982-0.995	-0.002	0.998	0.996-1

There was no collinearity between the independent variables.

**p < 0.01.

*p < 0.05. OR (95%CI) for statistically significant variables shown in bold font.

AP, Acute pancreatitis; PA, Physical activity; NAFLD, Nonalcoholic fatty liver disease.

**Table 4:** Multivariate logistic regression analysis of factors affecting the level of PA of patients with AP (model 2).

Variables	Low-level PA vs. high-level PA			Moderate-level PA vs. high-level PA			Low-level PA vs. moderate-level PA		
	B	OR	(95%CI)	B	OR	(95%CI)	B	OR	(95%CI)
(Intercept)	1.206			3.353			-1.311		
NAFLD	-1.399	0.247**	0.086-0.705	-1.114	0.328	0.078-1.383	-0.481	0.618	0.317-1.204
Biliary tract disease	0.14	1.151	0.441-2.999	-1.3	0.273*	0.078-0.955	0.618	1.856	0.972-3.543
Hypertension	0.073	1.076	0.291-3.98	1.097	2.996	0.427-21.011	-0.187	0.829	0.312-2.206
Diabetes	0.238	1.269	0.497-3.242	-0.196	0.822	0.23-2.938	0.273	1.313	0.615-2.803
No. previous AP attacks 0	Reference			Reference			Reference		
1	0.201	1.222	0.442-3.38	-0.299	0.742	0.188-2.933	-0.146	0.864	0.402-1.856
2	1.083	2.955	0.97-8.996	0.957	2.604	0.579-11.719	0.074	1.077	0.393-2.955
≥ 3	-1.587	0.205*	0.053-0.788	-1.219	0.295	0.055-1.599	-1.014	0.363*	0.154-0.855
None and Elementary (≤ 6 Y)	Reference			Reference			Reference		
Secondary (7–12 Y)	0.063	1.065	0.277-4.098	2.449	11.575**	1.965-68.165	-0.886	0.412	0.172-0.986
University (> 12 Y)	0.308	1.361	0.311-5.959	1.772	5.885	0.95-36.462	-0.885	0.413*	0.156-1.089
Staff of agencies and institutions	Reference			Reference			Reference		
Corporate employees	-0.473	0.623	0.095-4.109	-2.368	0.094	0.003-2.602	1.344	3.836	0.439-33.521
Commercial and service industry personnel	0.367	1.443	0.258-8.065	-1.593	0.203	0.009-4.554	1.594	4.923	0.593-40.879
Agricultural laborer	0.797	2.218	0.267-18.403	-1.255	0.285	0.009-9.063	1.843	6.313	0.603-66.045
Unemployed/Pensioner	-0.914	0.401	0.05-3.212	-1.813	0.163	0.006-4.401	1.282	3.604	0.396-32.814
Other job	0.457	1.58	0.233-10.699	0.78	2.181	0.073-65.576	0.59	1.804	0.182-17.92
Rural	Reference			Reference			Reference		
Urban	-0.79	0.454	0.18-1.144	-0.354	0.702	0.223-2.215	-0.101	0.904	0.441-1.852
Sedentary time	-0.01	0.99**	0.986-0.994	-0.014	0.987**	0.979-0.994	-0.107	0.899	0.775-1.042

In model 1 (**Table 3**), which used LPA as the control group, we showed that participants with NAFLD were 24.6% more likely to engage in HPA (OR, 0.246; 95%CI, 0.086–0.702), while those with more than three previous AP attacks had a 21.5% possibility (0.215, 0.057–0.817) of engaging in HPA and a 37.9% possibility (0.379, 0.162–0.885) of engaging in MPA compared to those without an AP attack. The participants with HPA also had a shorter ST (OR, 0.9; 95%CI, 0.986–0.994), while those with a secondary education degree were 42.4% more likely than those with no education or an elementary degree to engage in MPA (42.4, 95%CI 0.183–0.984). Using MPA as the control group, participants with secondary and education degrees were six times more likely to engage in HPA than those with no education or elementary degrees (OR, 6.903; 95%CI,1.481–32.187).

In model 2 (**Table 4**) after adjusting for the complications with LPA as the control group, we showed that NAFLD (OR, 0.247; 95% CI, 0.086-0.705), number of AP attacks more than three (OR, 0.205; 95% CI, 0.053-0.78), or a longer sedentary time (OR, 0.99; 95% CI, 0.986-0.994) all had a lower engagement in HPA. The number of AP attacks more than three (OR, 0.363; 95% CI, 0.154-0.855) and a university education degree (OR, 0.413; 95% CI, 0.156-1.089) were associated with a lower engagement in MPA. Using MPA as the control group, participants with biliary tract disease (OR, 0.273; 95%CI,0.078-0.955) and sedentary time (OR,0.987; 95%CI,0.979-0.994) had a lower engagement in HPA, while those with secondary and education degrees (OR,11.575; 95%CI,1.965-68.165) were more likely to engage in HPA than those with lower levels of education.

## 4 Discussion

This study represents the first comprehensive investigation into factors that may influence PA levels among adults with AP in Western China, incorporating demographic characteristics, health status, and lifestyle factors. The well-documented health benefits of PA include exercise-induced increases in interleukin-6 and -10 levels, which exert direct anti-inflammatory effects, while long-term exercise provides indirect anti-inflammatory benefits through improvements in body composition (Poorhabibi et al., 2025). Notably, a lack of exercise has been linked to insulin resistance and its associated metabolic syndrome and is considered to be a possible mechanism for the development of pancreatic disease (Małkowska, 2024). Publication of the research findings of a cohort study suggested that higher PA is associated with a lower risk of AP (Pang et al., 2018), while another study showed that PA also improved the quality-of-life, pain level, and disability conditions of patients with AP (Huang and Zhang, 2024). In addition, there is evidence that sedentary behaviors have distinct, independent associations with the deposition of adipose tissue (Malaikah et al., 2023), which in turn, affects the severity and pancreatic function of AP patients (Dong et al., 2024; Zhu et al., 2024). Taken together, these results indicate that PA should be emphasized as a critical factor in both the development and prognosis of AP.

In the current study, the number of participants with LPA was significantly higher than those with MPA or HPA, suggesting an overall lack of physical inactivity in the AP group. Interestingly, no significant differences in PA levels were observed according to age or sex, which may suggest a broader trend of physical inactivity in individuals at risk for developing AP. Our study showed that the ST of participants with AP was close to five hours, a duration longer than that reported in Southern Chinese (3.9 h) (Yu et al., 2018) and Eastern Chinese (4 h) individuals (Wang et al., 2022). The increase in ST has led to a corresponding increase in sedentary behaviors such as excessive use of electronic devices for work or play, which are known to be detrimental to public health with the potential to further reduce PA levels. Remarkably, this rising trend has also been reported in the more economically developed regions of China. Health promotion efforts for PA have traditionally focused on MPA and HPA (Matthews et al., 2016). However, an interesting finding of the current study was that higher levels of PA were associated with shorter ST in patients with AP. In contrast, previous studies have reported longer sedentary breaks in people in Brazil which correlated with better eating habits and higher levels of PA, even among sedentary teachers (Delfino et al., 2020), while higher levels of PA in Dutch adults increased the odds of total ST for ≥8 hr/day (Bakker et al., 2020). Similarly, another study reported no significant relationship between PA and ST (Wang et al., 2022). Taken together these results indicate that there is no strong or sufficiently established evidence that elevated PA levels lead to a longitudinal decrease in ST. These discrepancies are likely attributable to the methodological heterogeneity inherent in how sedentary behavior is conceptualized, measured, and classified across diverse populations. To minimize potential confounding effects from non-discretionary sedentary behaviors, we performed a sub-analysis focusing on our participants’ total sedentary time which consistently validated a significant association with PA. Collectively, our findings conform with the displacement hypothesis that suggests that screen time may be a more sensitive behavioral marker for lifestyle patterns than aggregate sedentary time, as it captures the specific trade-off individuals make between sitting and moving. Therefore, screen-based leisure may serve as a more granular and clinically relevant behavioral target than aggregate sedentary time alone. Our study demonstrated that screen time accounted for 68.3% of all sedentary behaviors in the participants, a proportion which was statistically significant. We also observed a notable positive correlation between overweight status and the amount of time spent online or playing video games in middle-aged adults (≥ 45 yr). Participants who successfully maintained weight loss reported spending less time sitting compared to obese individuals with stable weights. This suggested that reduced sedentary time may contribute to weight management and potentially lower the risk of developing AP (Roake et al., 2021). There is also evidence that watching television or videos is more prevalent in older adults (Su et al., 2023). Findings from studies in the United States (Yang et al., 2019) and Canada (Prince et al., 2020a) also reported that total leisure screen time appeared to have increased significantly with the use of computers and electronic devices. Previous reports also noted that reading of printed materials such as books, newspapers, and magazines is the only sedentary activity that correlates positively with beneficial outcomes such as the maintenance of academic performance and cognitive functioning (Gogniat et al., 2025). However, less than 7.6% of the patients with AP in the current study self-reported reading as a sedentary behavior. Understanding these specific domains and types of information will enable the design of individually directed interventions to achieve the key goal of behavior modification in people at risk of developing AP.

Using LPA as the control group, we showed that individuals with NAFLD were 24.6% more likely to engage in HPA compared to participants without NAFLD. Similarly, a study of a representative sample from the U.S. reported that compared to individuals without NAFLD, those with NAFLD tended to be older, male, had a higher BMI, performed less PA, and were more likely to have diabetes and the metabolic syndrome (Heredia et al., 2022). This trend may be influenced by the quality of the diet, as a multiethnic cohort study has demonstrated an inverse relationship between diet quality and the prevalence of NAFLD (Yoo et al., 2020). The challenge we face is that 31.9% of AP patients in our study had NAFLD. Due to the insidiousness nature of NAFLD, with a lack of obvious symptoms in the early stages, chronic progression, and a lack of specific screening methods, the prevalence of this disorder may be underestimated. Early detection and measures such as lifestyle interventions are therefore more difficult to achieve. NAFLD is associated typically with obesity, insulin resistance, diabetes, and dyslipidemia, with the accumulation of fat accompanied by inflammation and injury that promotes cell senescence in both human adipose and liver cells (Wang et al., 2021; Spinelli et al., 2023). Chronic inflammation may also cause physical discomforts such as fatigue and lethargy, which directly affects an individual’s ability to be physically active. In addition, people with NAFLD may have reduced muscle mass and muscle strength, which directly affects their ability to be physically active (Cai et al., 2020). Furthermore, there is evidence of a higher prevalence of comorbid psychiatric disorders such as coexisting depression, anxiety and/or stress in adults with NAFLD, which influences their motivation to engage in PA (Shea et al., 2024). Overall, physical inactivity may contribute to the development of NAFLD, while NAFLD itself may perpetuate physical inactivity, thereby creating a vicious cycle.

Furthermore, our analyses stratified according to severity and etiology showed no significant differences in PA levels across severity categories or etiology groups. This indicated that PA levels were not associated with acute-phase clinical indicators in this cross-sectional setting (**Supplementary Table 2**). This negative result may be attributed to the fact that PA reflects long-term lifestyle habits, whereas acute-phase laboratory indicators and severity are influenced by immediate pathophysiological changes, leading to temporal misalignment. In addition, the relatively small sample size in certain of our subgroups may have limited the statistical power to detect subtle differences. These findings suggest that exercise prescriptions for AP patients should be tailored based on stable clinical factors such as recurrence history and NAFLD status, rather than acute-phase biomarkers. An additional unexpected finding in our study was that pre-onset BMI was not associated significantly with PA levels in AP patients (*P* = 0.244), a finding which contrasts with patterns observed in the general population. We propose that this decoupling can be explained by etiological diversity. First, young, obese patients with hypertriglyceridemia often maintain normal or even high PA levels before an attack, whereas older, lean patients with biliary stones tend to be sedentary due to aging. This clinical pathological mixture may obscure the expected negative association between BMI and PA. Second, in hypertriglyceridemic AP, dietary patterns may exert a stronger influence on BMI than PA regulation. Third, subclinical symptoms (e.g., biliary colic) before an attack may lead to a reduction in spontaneous activity across all BMI groups. To further investigate this possibility, we performed a stratified analysis by etiology (**Supplementary Table 3**), which confirmed that BMI distribution differed significantly across etiologies (χ² = 54.275, *P* < 0.001). Specifically, 81.2% of hypertriglyceridemic AP patients were overweight/obese, whereas only 43.3% of biliary AP patients had these two conditions. This decoupling suggests that interventions to prevent PA should not be limited to obese individuals. Normal-weight but sedentary individuals also have a high risk of AP and must be included in preventive health counseling.

Our findings that NAFLD is associated with lower odds of HPA, indicates that promoting PA levels in individuals with NAFLD is essential. Interventions could start by improving the quality of their diet, given its role in NAFLD pathogenesis and overall energy metabolism. As measured by our FFQ, diet quality refers to a dietary pattern rich in fruits, vegetables, and low-fat dairy, with limited consumption of high-fat meats and processed foods, this finding may help researchers further explore the public health perceptions of groups with different health conditions and develop individual cultural public health recommendations and interventions. Notably, our study was based on cross-sectional analyses, and therefore it was not possible to confirm whether those individuals with impaired liver and pancreas function were able to engage in adequate PA and eat a healthy and diverse diet, or whether they had already changed their behavior in response to their symptoms or a diagnosis.

This study indicated that concomitant biliary tract disease significantly influences the level of HPA prior to the onset of AP. This association can be explained by behavioral and pathophysiological mechanisms. Firstly, this includes recurrent abdominal pain and indigestion caused by gallstones or chronic cholecystitis or high-intensity or prolonged exercise that often exacerbates gastrointestinal symptoms (Al-Beltagi et al., 2025), leading patients to actively reduce strenuous exercise to avoid triggering an exacerbation of pain. Secondly, the relationship between biliary tract diseases and reduced PA is likely to be bidirectional. A lack of PA is a proven risk factor for the formation of gallstones (Ye et al., 2024). Conversely, the lower levels of PA observed prior to the onset of biliary acute pancreatitis may reflect a long-term sedentary lifestyle that initially led to cholestasis. The current study therefore highlights the clinical importance of developing personalized PA guidelines for patients diagnosed with biliary tract disease that are tailored to their symptom tolerance, as one of the measures to prevent secondary acute pancreatitis. However, our results show that there was a lack of correlation between PA and clinical markers in the acute phase based on the severity and etiology of this phase. These negative results are likely to be due to the time mismatch between long-term PA habits and acute pathophysiological changes, with the limited statistical power of the small sample subgroups making the situation even more complicated.

In addition, people with AP with more than three previous episodes of the disorder were more likely to engage in LPA than those with no previous AP attack. Pancreatitis is a progressive inflammatory disease of the pancreas, and repeated episodes can lead to a loss of gland function, resulting in complications such as malabsorption, diabetes mellitus, and severe abdominal pain. These issues often contribute to malnutrition, osteoporosis, and sarcopenia (Kuan et al., 2021). A systematic review carried out in 2015 of high-quality cohort studies with at least one year of follow-up showed that recurrent AP developed in 21% of patients after the first episode of the disorder (Sikora Kessler et al., 2026). The diminished body function and endurance associated with AP reminded these patients of the realities of losing control of disease progression, leading to suppression of further development to achieve their life goals, followed by psychological problems. For example, the prevalence of depression in SAP patients can be up to 34.3% (Wang et al., 2023). Physical and psychological disturbances may therefore be potential barriers to moderate PA in AP patients, although we did not directly assess this relationship in our study. However, currently we do not have sufficient evidence to provide recommendations for an optimal PA program in patients with AP. Therefore, we emphasize there is a need to urgently investigate the PA of AP patients as a basis for intervention. The current study demonstrated that the education level of an individual was a critical factor that affected PA. An interesting finding was that high literacy promoted the occurrence of HPA in the medium-high level group, whereas in the low-middle level group, high literacy reduced the production of HPA. In a Portuguese study, a ‘higher risk’ behavior pattern (i.e., low PA/high sedentary time) was present in 37.3% of adults and was likely associated with having 12 or more years of education (Martins et al., 2021). The Latin American Nutrition and Health Study (LANHS) (Ferrari et al., 2020) also reported that participants with higher education levels exhibited the lowest levels of moderate-to-vigorous PA, which aligns with our observation that higher literacy may reduce occupational PA in certain subgroups. A possible reason for this finding is that people with no elementary education are more likely to be older than people with a secondary degree or above. Higher literacy levels result in a greater availability of career resources with occupational activities being more likely mental than physical (Prince et al., 2020b). However, in highly educated groups, HPA also tends to compensate for a lack of daily PA. Therefore, according to the education level and occupational characteristics of AP patients, it is important to help balance their daily lives, break through the dilemma limitations through professional knowledge, and achieve self-adaptation from knowledge guidance to motivation stimulation. Since performing different levels of PA to reduce the risk of AP still needs to be confirmed, the exercise prescription for people with different characteristics should be improved, and patients with AP should be encouraged to carry out more diversified recreational PA to improve their ability to prevent disease.

This study identified the characteristics of key factors associated with PA levels in a specific clinical group of AP patients from a tertiary hospital setting. The strength of this research lies in the scarcity of similar studies on AP patients in China, and offers new insights into the epidemiology of this population, thereby enabling the development of more targeted health promotion programs. Several limitations of the study need to be acknowledged. First, the findings are from a single center and as there are regional differences in lifestyle and dietary habits our findings may not be generalizable to the broader community-based AP population. The study was also a cross-sectional design that was not able to assess the causal relationship between the influencing factors and PA levels. Second, although we did not distinguish between occupational and leisure-time screen use, we consider that total screen time remains a meaningful metric. The metabolic risk associated with sedentary behavior is primarily a function of cumulative time, and therefore establishing a clear, total duration target will serve as a practical tool for clinicians to provide consistent lifestyle guidance and improve patient compliance. Third, although we controlled for key confounders, certain unmeasured variables including rare genetic factors, drug history, and minor trauma could not be fully accounted for. Therefore, our results represent epidemiological associations rather than proof of definitive causality. Finally, the reliance on self-reported PA via the IPAQ long form may have introduced recall bias, particularly given the low PA levels in this population. Future research should therefore use longitudinal or interventional designs to establish causal relationships between identified factors (e.g., NAFLD, AP recurrence) and PA levels. Multi-center studies across diverse regions in China are also needed to enhance generalizability. Moreover, objective measures of PA (e.g., accelerometers) should be considered to reduce recall bias, and investigations into tailored exercise prescriptions for AP patients with comorbidities like NAFLD are warranted.

## 5 Conclusion

This study investigated factors that influenced PA levels in adults with AP with the aim of addressing the gap in the current data. The findings highlight a higher frequency of physical inactivity in AP patients in Western China that is influenced by factors such as ST, NAFLD, biliary tract disease, education level, and the number of AP episodes. Specifically, our results demonstrated that prolonged sedentary time, particularly screen time, was prevalent and inversely associated with PA levels. The presence of NAFLD and a history of recurrent AP episodes were strongly associated with lower PA, which was likely due to compounded physical discomfort, fatigue, and psychological burdens. Furthermore, education level emerged as a significant sociodemographic determinant, with complex relationships observed across different educational strata.

These insights support the need for individualized exercise prescriptions (e.g., tailored recommendations on the type, frequency, intensity, and duration of PA based on a patient’s specific condition, comorbidities, recurrence history, and education level, with targeted health education programs tailored to different stages of AP. Interventions should also prioritize reducing sedentary behaviors, especially recreational screen time, while promoting feasible and enjoyable PAs. For patients with NAFLD, biliary tract disease or recurrent AP, integrated care approaches addressing both physical and psychological barriers to activity are essential. Future longitudinal and interventional research should focus on establishing causal relationships, determining optimal PA levels, and evaluating the impact of tailored exercise programs on health outcomes across the spectrum of pancreatic diseases to guide systematic and effective health interventions.

## Data Availability

The original contributions presented in the study are included in the article/**Supplementary Material**. Further inquiries can be directed to the corresponding author.
