# Supplementary material for: Analysis of factors associated with physical activity in patients with acute pancreatitis: a cross-sectional study in China
Source: Front Physiol. 2026 Jul 29;17:1786484. doi: 10.3389/fphys.2026.1786484 (PMC13461537; doi:10.3389/fphys.2026.1786484)
Supplement: Supplementary file 1 [file Table1.docx]

**Supplementary Data**

Table S1. Association between screen time and outcomes.

| Variables | Low Screen Time (< 4 h/d, n = 218) | High Screen Time (> 4 h/d, n = 190) | χ2 | P-value |
| --- | --- | --- | --- | --- |
| Physical Activity Level, n (%) |  |  | 23.107 | < 0.001 |
| Low | 145 (47.9%) | 158 (52.1%) |  |  |
| Moderate | 38 (58.5%) | 27 (41.5%) |  |  |
| High | 35 (87.5%) | 5 (12.5%) |  |  |
| NAFLD, n (%) |  |  | 1.353 | 0.245 |
| No | 154 (55.4%) | 124 (44.6%) |  |  |
| Yes | 64 (49.2%) | 66 (50.8%) |  |  |
| No. previous AP attacks, n (%) |  |  | 2.698 | 0.441 |
| 0 | 102 (49.8%%) | 103 (50.2%) |  |  |
| 1 | 41 (56.9%) | 31 (43.1%) |  |  |
| 2 | 20 (52.6%) | 18 (47.4%) |  |  |
| ≥3 | 55 (59.1%) | 38 (40.9%) |  |  |

Table S2. Stratified analysis of the association between BMI and Physical Activity (PA) by recurrence status.

| Variables | LPA | MPA | HPA | χ2 | P-value |
| --- | --- | --- | --- | --- | --- |
| Severity |  |  |  | 1.952 | 0.745 |
| mild | 144 (75.0%) | 31 (16.1%) | 17 (8.9%) |  |  |
| moderately severe | 55 (77.4%) | 8 (11.3%) | 8 (11.3%) |  |  |
| severe | 104 (71.7%) | 26 (17.9%) | 15 (10.4%) |  |  |
| Etiology |  |  |  | 4.534 | 0.605 |
| biliary | 92 (68.7%) | 26 (19.4%) | 16 (11.9%) |  |  |
| Hypertriglyceridemia | 105 (76.1%) | 20 (14.5%) | 13 (9.4%) |  |  |
| Alcohol | 28 (71.8%) | 7 (17.9%) | 4 (10.3%) |  |  |
| Others | 78 (80.4%) | 12 (12.4%) | 7 (7.2%) |  |  |

Table S3. Stratified analysis of the association between BMI and physical activity by etiology.

| Variables | Normal and underweight (BMI < 24.0, kg/m^2^) | Overweight/Obesity (BMI ≥ 24.0, kg/m^2^) | χ2 | P-value |
| --- | --- | --- | --- | --- |
| Etiology |  |  | 54.275 | < 0.001 |
| Biliary | 76 (56.7%) | 58 (43.3%) |  |  |
| Hypertriglyceridemia | 26 (18.8%) | 112 (81.2%) |  |  |
| Alcohol | 12 (30.8%) | 27 (69.2%) |  |  |
| Others | 56 (55.7%) | 41 (42.3%) |  |  |
